# Effects of (S)-3,4-DCPG, an mGlu8 receptor agonist, on hippocampal long-term potentiation at perforant pathway-dentate gyrus synapses in prenatal valproic acid-induced rat model of autism

**DOI:** 10.1038/s41598-024-63728-y

**Published:** 2024-06-07

**Authors:** Parsa Gholipour, Zahra Ebrahimi, Reihaneh Mohammadkhani, Reza Ghahremani, Iraj Salehi, Abdolrahman Sarihi, Alireza Komaki, Seyed Asaad Karimi

**Affiliations:** 1 Neurophysiology Research Center, Hamadan University of Medical Sciences, Shahid Fahmideh Street, Hamadan, 65178/518 Iran; 2 Department of Exercise Physiology, Faculty of Sport Sciences, University of Birjand, Birjand, Iran; 3 Department of Neuroscience, School of Science and Advanced Technologies in Medicine, Hamadan University of Medical Sciences, Hamadan, Iran

**Keywords:** Autism spectrum disorder, Valproic acid, Long-term potentiation, mGlu8 receptor, Hippocampus, Long-term potentiation, Membrane potential, Disease model

## Abstract

Autism spectrum disorder (ASD) is a pervasive neurodevelopmental condition characterized by social interaction deficits, communication impairments, repetitive behaviors, and sensory sensitivities. While the etiology of ASD is multifaceted, abnormalities in glutamatergic neurotransmission and synaptic plasticity have been implicated. This study investigated the role of metabotropic glutamate receptor 8 (mGlu8) in modulating long-term potentiation (LTP) in a rat model of ASD induced by prenatal valproic acid (VPA) exposure. To induce an animal model with autism-like characteristics, pregnant rats received an intraperitoneal injection of 500 mg/kg of sodium valproate (NaVPA) on embryonic day 12.5. High-frequency stimulation was applied to the perforant path-dentate gyrus (PP-DG) synapse to induce LTP, while the mGlu8 receptor agonist (S)-3,4-dicarboxyphenylglycine (DCPG) was administered into the DG. The results revealed that VPA-exposed rats exhibited reduced LTP compared to controls. DCPG had contrasting effects, inhibiting LTP in controls and enhancing it in VPA-exposed rats. Moreover, reduced social novelty preference index (SNPI) in VPA-exposed rats was reversed by intra-DG administration of S-3,4-DCPG. In conclusion, our study advances our understanding of the complex relationship between glutamatergic neurotransmission, synaptic plasticity, and VPA-induced autism model. The findings suggest that mGlu8 receptor dysfunction plays a role in the impaired synaptic plasticity seen in ASD.

## Introduction

Autism spectrum disorder (ASD) represents a pervasive neurodevelopmental condition marked by deficits in social interaction, language and communication impairments, repetitive and stereotypical behaviors, limited interests and activities, and occasionally heightened sensitivities to sensory stimuli such as sound or touch, along with potential displays of aggression^1–3^.

The origin of ASD is intricate and diverse, involving a combination of genetic, epigenetic, or environmental factors either independently or in combination^4^. ASD does not have a single identifiable cause, and the majority of cases are of unknown origin^5^.

Abnormality in glutamatergic neurotransmission has been suggested in the pathophysiology of autism^6,7^. Increased serum levels of glutamate in adult patients with autism has been reported by Hashimoto et al.^6^, and Fatemi proposed a hyperglutamatergic hypothesis of autism^8^. Unlike hyperglutamatergic hypothesis, hypoglutamatergic theory^9^ has recently been proposed, based on studies of glutamate receptor dysfunction and pharmacological effects of glutamatergic agonists and antagonist. Moreover, changes have been observed in ionotropic (iGlu) and metabotropic (mGlu) glutamate receptors expression in autism^10^. iGlu receptors are ligand-gated ion channels and have been divided into four subtypes including: AMPA receptors, kainate receptors, NMDA receptors and delta receptors^11^. The mGlu receptors are G-protein coupled receptors and have eight subtypes and are classified into three groups including group I (mGlu1 and mGlu5), group II (mGlu2 and mGlu3), and group III (mGlu4, mGlu6, mGlu, and mGlu8) depending on their signal transduction pathways, sequence homology, and pharmacological selectivity. The group III of mGlu receptors are coupled with the Gi/o proteins^12^.

Recent research findings suggest that dysfunction in mGlu receptors is associated with ASD^7^. For instance, disrupted signaling of mGlu5 receptors appears to be a shared feature across various ASD subtypes^13–16^. Moreover, Group I and group II mGlu receptors are upregulated in the synapses of infant rats prenatally exposed to valproic acid (VPA)^17^. These findings highlight the potential significance of mGlu receptor-related pathways in understanding and potentially treating ASD.

Changes in synaptic plasticity and local and distant connectivity in the brain have been proposed as a possible cause of autistic behavior^18,19^, and dysregulation of synaptic plasticity precedes appearance of morphological defects in autism^20^. In addition, impairments of synapse formation and synaptic plasticity^21,22^, which ultimately lead to functional and cognitive impairments, are thought to be major contributors to ASD pathology. Synaptic plasticity occur locally in individual synapses and include long-term potentiation (LTP) or long-term depression (LTD)^23,24^. Previous studies have shown that hippocampal LTP, a biological model for learning and memory, is attenuated at the medial perforant path-dentate gyrus (PP-DG) synapse in the animal model of ASD^20,25^. But its underlying mechanism (s) has not yet been determined.

It is established that the mGlu receptors are critically required for synaptic plasticity^26^. Association between mGlu8 receptors and autism provided the rationale for testing mGlu8 receptors agonist in ASD. Expression of mGlu8 receptors in the CNS has been observed at the presynaptic level in the cerebellum, olfactory bulb, hippocampus, and cortical areas^27^. Also, perforant path inputs to the DG express high levels of mGlu8 receptors^28,29^. Serajee et al. have suggested a possible association between mGlu8 receptors and autism^30^. mGlu8 receptors negatively modulate glutamate transmission and thus serve to prevent pathological changes in neuronal hyperexcitability and homeostasis^31^. Thus, dysfunction of the mGlu8 receptors may result in neuronal damage.

Synthesis of a potent and selective mGlu8 receptor agonist (S)-3,4-dicarboxyphenylglycine (DCPG)^32^, has led to an increase in interest into the possible functional role of this receptor within the central nervous system (CNS). Because synaptic plasticity relies on the integrity of glutamatergic transmission that could be compromised by ASD, we hypothesized that mGlu8 receptors may be involved in LTP deficiency in animal models of autism. Therefore, the goal of the current study was to assess the involvement of in intra-DG mGlu8 receptor in the induction of LTP the perforant path to granule cell synapse in the DG of the hippocampus in offspring of a rat model of autism induced by prenatal exposure to VPA.

## Methods

### The VPA rat model of autism

All experimental protocols were approved by the Ethics Committee of Hamadan University of Medical Sciences (IR.UMSHA.REC.1397.936) and were conducted in accordance with the National Institutes of Health Guide for the Care and Use of Laboratory Animals. All experimental procedures have been done after proper animal handling to minimize stress. For pregnancy, two female Wistar rats were mated overnight with one sexually mature male rat (i.e., 6 weeks of age) of the same strain. The presence of a vaginal plug or sperm in the vaginal smear the following morning confirmed coition on embryonic day 1. To induce a rat model of autism, sodium valproate (NaVPA, Sigma, UK) was dissolved in normal saline to a concentration of 150 mg/ml (pH 7.3). On E12.5, VPA-dams received a single intraperitoneal (i.p.) injection of NaVPA (500 mg/kg, 3.3 ml/kg); control groups received a single injection of saline as vehicle (i.p., 3.3 ml/kg). Animals were kept at a room temperature of 23 ± 3 °C with a 12:12 h light/dark cycle, and were given free access to food and tap water. Dams were housed individually and allowed to raise their own litters. The offspring were used for LTP recording. This study is reported in accordance with ARRIVE guidelines.

### Social interaction test

Social interaction impairment stands as a predominant and consequential feature within cases of autism. To investigate this, we did a three-chamber sociability and social novelty test between postnatal days 37 and 40. The experimental setup involved a box measuring 114 cm × 51 cm × 51 cm, featuring three chambers of equal length. These chambers were partitioned by transparent Perspex walls with central openings, facilitating unrestricted movement between them. After a 5-min initial habituation period with the empty box, the test rat was introduced to the 10 min "sociability" session. During this session, it encountered an unfamiliar intruder placed in a wire cage measuring 12 cm × 18 cm × 12 cm, while the other wire cage remained empty in the opposite chamber. During the third phase, the social novelty preference test was conducted, wherein the empty cage chamber was substituted with a new, unfamiliar male rat that had never before interacted with the test rat. This social novelty preference test aimed to evaluate the test animal’s inclination to spend more time with the unfamiliar rat compared to the familiar one. Each animal underwent a 10-min social novelty test. All behaviors were tracked using a Maze Router homemade software. Finally, the Social Novelty Preference Index (SNPI) was calculated as an indicator of social interaction. The SNPI was determined by calculating the ratio of time spent on the novel side over the time spent on the familiar side. The selective mGlu8 receptor allosteric agonist (S-3,4-DCPG) was administered at dose 1 µM/0.5 μl saline per side into the DG through implanted cannulas on both sides before the sociability and social novelty preference sessions.

### Cannulation surgery and intra-DG injection

The rats were anesthetized using a combination of Ketamine/Xylazine (K: 100 mg/kg; X: 10 mg/kg) and placed in a stereotaxic device (Stoelting, USA). A heating pad was used to maintain the temperature of the animals at 36.5 ± 0.5 °C. An incision was made to expose the rats' skull, and two points were determined and drilled into the skull at stereotaxic coordinates of AP: − 3.8 mm from bregma and ML: + 2.3 mm from the midline along the sagittal suture. Two guide cannulas (23-Gauge) with a length of 12 mm were inserted into the holes, targeting the DG at a depth of 2.7 mm from the top of the skull, following the atlas of the rat brain (Paxinos and Watson, 2006). The guide cannulas were anchored with a jeweler's screw, and the incision was closed with dental cement. After surgery, dummy inner cannulas, extending 0.3 mm beyond the guide cannulas, were inserted into the guide cannulas and left in place until injections were made. All rats were allowed to recover for one week before starting behavioral testing. For intra-DG injection, the rats were gently restrained by hand, and the dummy guide cannulas were removed from the guide cannulas. S-3,4-DCPG or saline was directly injected into the DG through the guide cannulas using injector cannulas (30-gauge) positioned 0.5 mm below the tip of the guide cannulas. Polyethylene tubing (PE-20) was used to attach the injector cannula to the 5-μL Hamilton syringe. S-3,4-DCPG was administered bilaterally into the DG, with a volume of 0.5 μl per side. Bilateral DG injections were performed over a 120 s period, and the injection cannulas were left in the guide cannulas for an additional 120 s to facilitate drug delivery. (S)-3,4-Dicarboxyphenylglycine (S-3,4-DCPG) (Tocris, UK), a selective mGlu8 receptor allosteric agonist, was dissolved in normal saline (0.9% NaCl). Control groups received saline.

### Electrode implantation surgery

On postnatal day 45–55, rats were anesthetized with urethane, and placed into a stereotaxic apparatus for surgery, electrode implantation and field potential recording. The methodologies used in this section were similar to prior studies that published by our laboratory^33–35^. Briefly, under urethane anesthesia induced by intraperitoneal injection (1.5 g/kg), rats head was fixed in a stereotaxic apparatus for surgery and recording. A heating pad was used to maintain the temperature of the animals at 36.5 ± 0.5 °C. Small holes were drilled in the skull. Afterwards, two bipolar electrodes, made of stainless steel with Teflon cover (125 µm diameter, Advent Co., UK) were positioned in the right cerebral hemisphere. The stimulating electrode was placed in the PP [AP: − 8.1 mm from bregma; ML: + 4.3 mm from midline; DV: 3.2 mm from the skull surface], an infusion cannula was glued to the recording electrode and then lowered into the dentate gyrus (DG)^36^ [AP: − 3.8 mm from bregma; ML: + 2.3 mm from midline; DV: 2.7–3.2 mm from the skull surface] according to the Paxinos and Watson atlas of the rat brain^34,37^. The electrodes were lowered very slowly (0.2 mm/min) from cortex to the hippocampus, in order to minimize trauma to the brain tissue. S-3,4-DCPG, was administered into the DG over 5 min at concentrations of 1 µM/0.5 μl))^29^. Control groups received saline.

### Electrophysiological recordings and LTP induction

Input–output current profiles were obtained by stimulating the PP to determine the stimulus intensity to be used in each animal. Single 0.1 ms biphasic square wave pulses were delivered through constant current isolation units (A365 WPI) at a frequency of 0.1 Hz.

**Figure 1:**
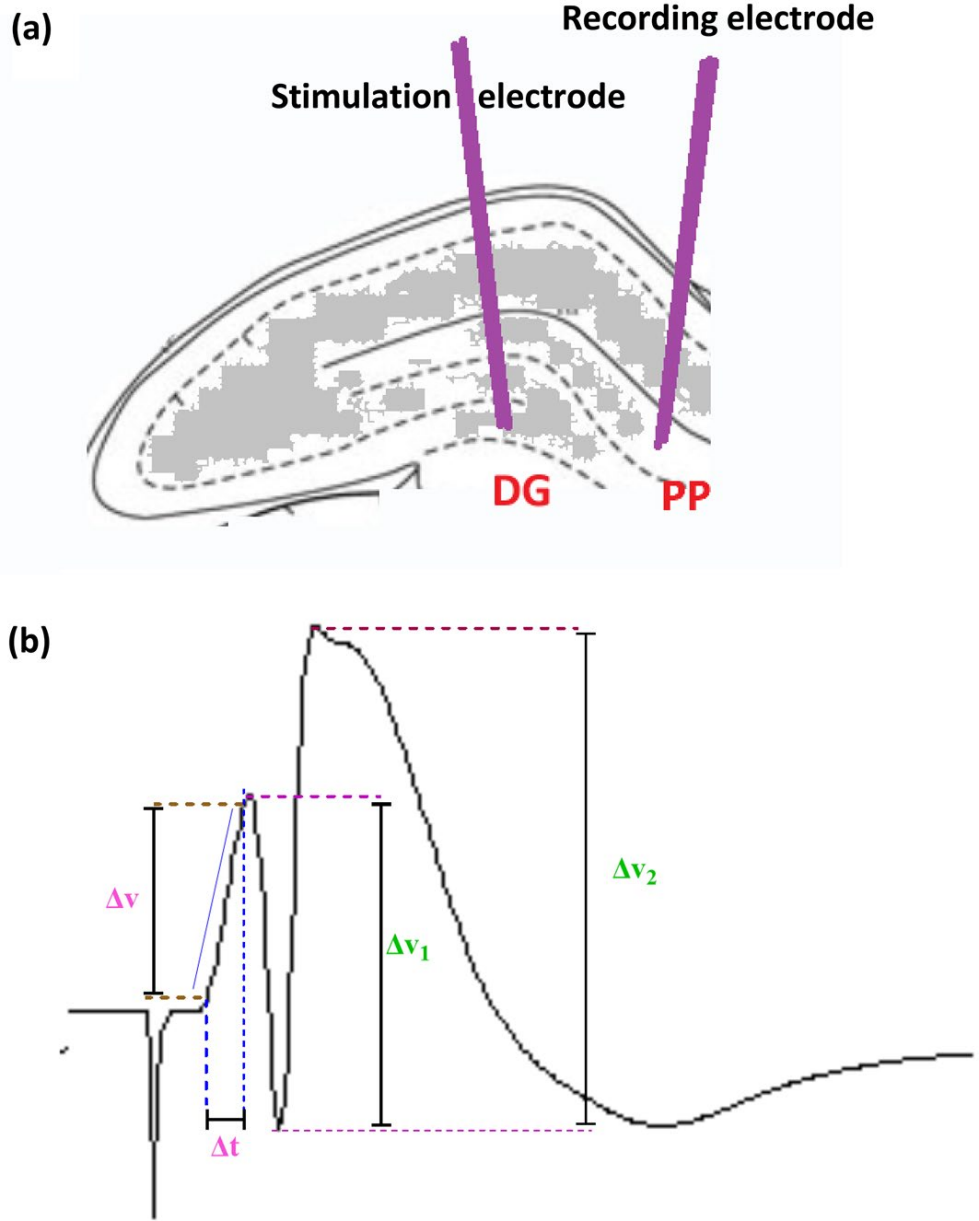
Schematic diagram of electrode locations (**a**), and measurement of evoked potentials (EPSP slope and PS amplitude) in the hippocampus (**b**). EPSP slope and PS amplitude were calculated using Eqs. 1 and 2, respectively (refer to the text for details). Here, ΔV represents the potential difference, and ΔT signifies the time difference.

The field potential recordings were obtained in the granular cells of the DG following stimulation of the PP (Fig. 1a). Test stimuli were delivered to the PP every 10 s. Electrodes were positioned to elicit the maximum amplitude of population spike (PS) and field excitatory post synaptic potentials (fEPSP). After ensuring a steady state baseline response, which was taken about 40 min, LTP was induced using a high frequency stimulation (HFS) protocol of 400 Hz (10 bursts of 20 stimuli, 0.2 ms stimulus duration, 10 s interburst interval) at a stimulus intensity that evoked a PS amplitude and field EPSP slope of approximately 80% of the maximum response. Both fEPSP and PS were recorded 5, 30, and 60 min after the HFS in order to determine any changes in the synaptic response of DG neurons. For each time-point, 10 consecutive evoked responses were averaged at 10 s stimulus interval^38–40^.

For stimulations, the parameters of the stimuli were defined in homemade software and were sent via a data acquisition board linked to a constant current isolator unit (A365 WPI, USA) prior delivery to the PP through the stimulus electrodes. The induced field potential response from the DG, was passed through a preamplifier, then was amplified (1000×) (Differential amplifier DAM 80 WPI, USA), and filtered (band pass 1 Hz to 3 kHz). This response was digitized at a sampling rate of 10 kHz, and was observable on a computer (and an oscilloscope). It was saved in a file to facilitate later offline analysis.

### Measurement of evoked potentials

The evoked field potential in the DG has two components: PS and fEPSP. During electrophysiological recordings changes in PS amplitude and fEPSP slope were measured^34^.

PS amplitude and EPSP slope were calculated according to Eqs. (1) and (2), respectively (see Fig. 1b).

1 $$EPSP = \frac{\Delta V}{{\Delta T}}$$

2 $$PS = \frac{{\Delta V_{1} + \Delta V_{2} }}{2}$$

where (see Fig. 1b); ΔV = the potential difference between points c and d; ΔT: Time difference between points a and b; ΔV1 = the potential difference between points e and f; ΔV2 = the potential difference between points f and g.

### Statistical analysis

Data were expressed as the mean ± standard error of mean (SEM) and were processed by commercially available software GraphPad Prism® 8.0.2. The data normality test was performed using Shapiro–Wilk test. Data were analyzed by two-way repeated measures ANOVA followed by Bonferroni post-test. Additionally, one-way ANOVA analysis was employed to assess the average potentiation of EPSP slope and PS amplitude between experimental groups. LTP data were normalized to the mean value of fEPSP slopes and PS amplitude recorded prior to the induction of LTP (Eq. 3)^41^, and reported as mean ± SEM. A probability of 0.05 was considered as the criterion for significance. A two-way ANOVA analysis was used for the SNPI data.

3 $${\text{LTP}} = \frac{{{\text{the\,EPSP\,or\,PS\,value\,after\,HFS\,induction }} \times 100{\text{\% }}}}{the\,average\,EPSP\,or\,PS\,at\,baseline}$$

### Ethical approval

All animal experimental procedures were performed in accordance with the guidelines for proper conduct of animal experiments issued by the Ethics Committee of the Hamadan University of Medical Sciences and performed according to the ‘Guide for the Care and Use of Laboratory Animals’, prepared by the National Academy of Sciences and published by the National Institutes of Health (NIH publication 86-23 revised 1985). This study is reported in accordance with ARRIVE guidelines.

## Results

### Effects of DCPG on social interaction behaviour

**Figure 2:**
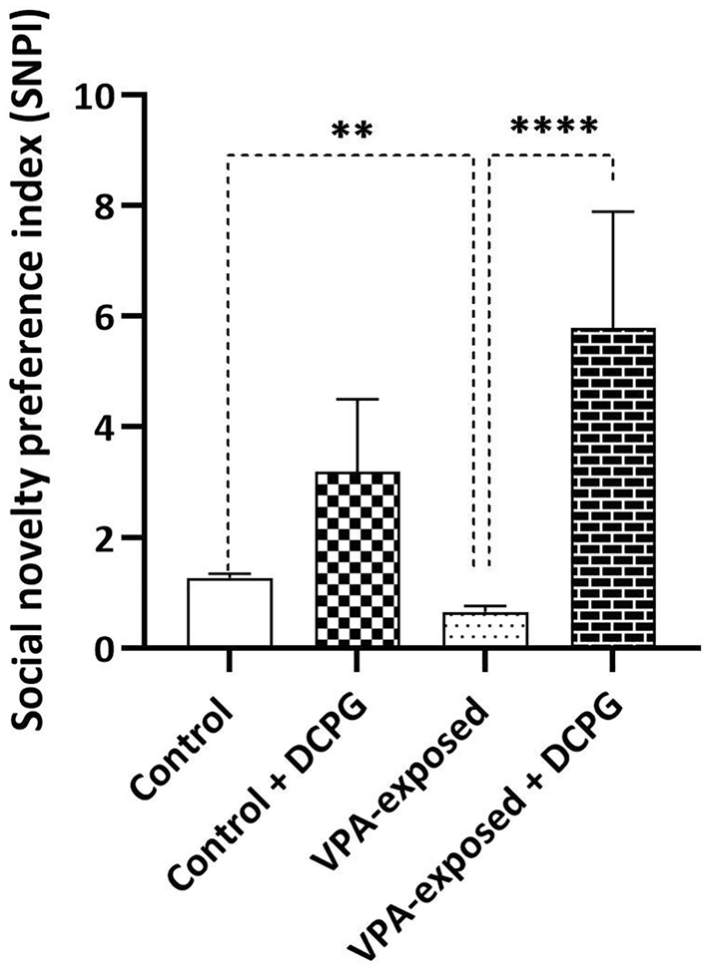
Reduced social novelty preference index (SNPI) in VPA-exposed rats was reversed by intra-DG administration of S-3,4-DCPG. Data are presented as mean ± SEM. **P < 0.01, ****P < 0.0001 (two-way ANOVA). The number of animals in each group for social interaction was as follows: Control group: 9 rats, VPA-exposed group: 10 rats, Control + DCPG group: 8 rats, VPA-exposed + DCPG group: 9 rats.

As we previously reported, VPA injection led to notable changes in the social interaction patterns of offspring rats. In this study, offspring rats born from dams treated with VPA on E12.5 were assessed for sociability between Postnatal Days 37–40, revealing evident impairments in Social Novelty Preference Index (SNPI). SNPI serves as a valuable metric for assessing social interaction in experimental settings, particularly in studies involving animal models of neurodevelopmental disorders such as autism. Two-way ANOVA analysis revealed a significant decrease in SNPI following VPA exposure. Furthermore, the intra-DG administration of S-3,4-DCPG (1 µM/0.5 μl) significantly reversed the decrease in SNPI induced by VPA exposure, with no significant effect in control animals (DCPG effect [F (1, 32) = 18.13, P = 0.0002], Fig. 2).

### Effects of DCPG on field EPSP LTP in PP-DG pathway

**Figure 3:**
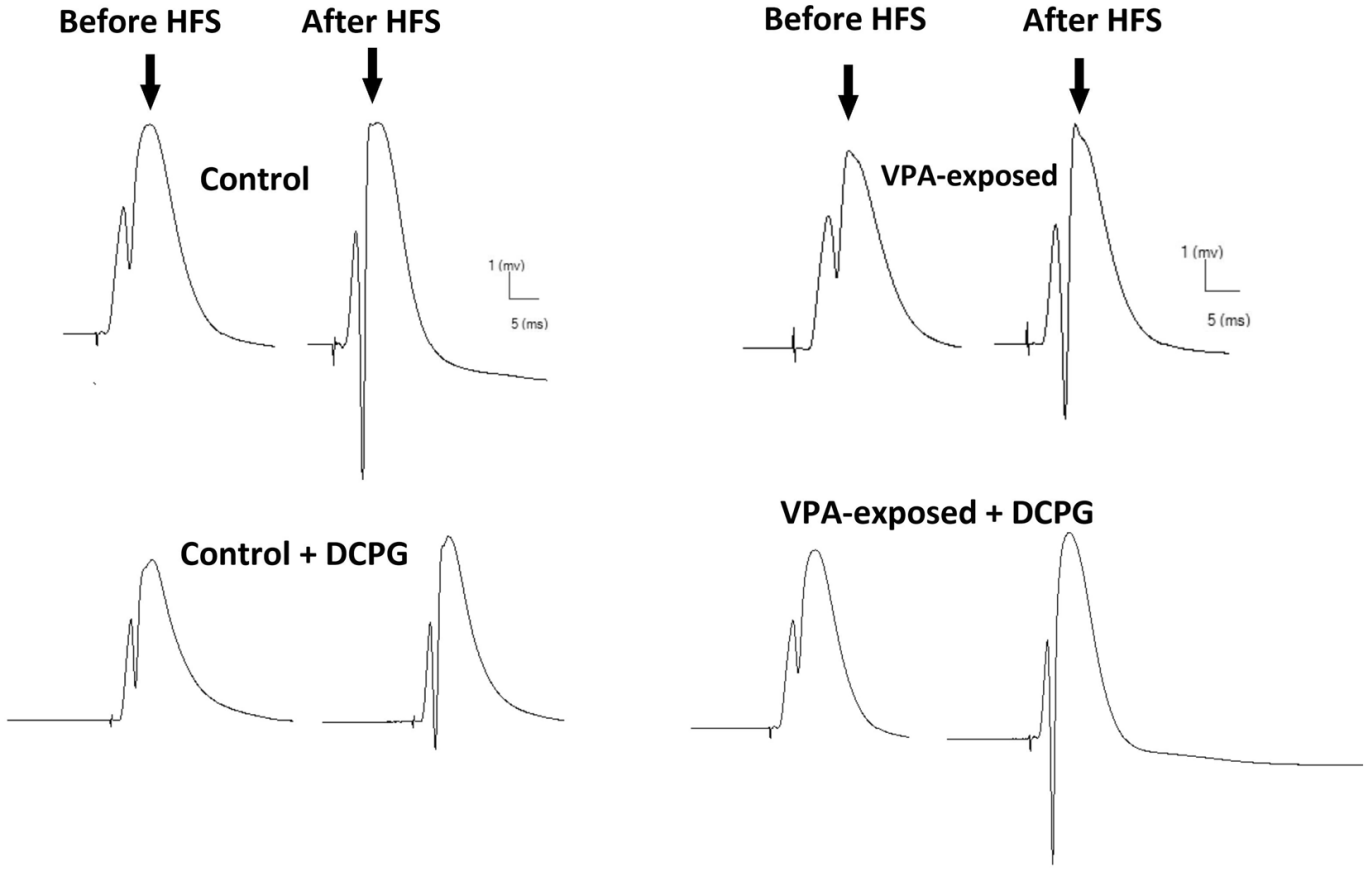
Representative sample traces of evoked field potential in the DG recorded prior to and 60 min after high‑frequency stimulation in all groups.

**Figure 4:**
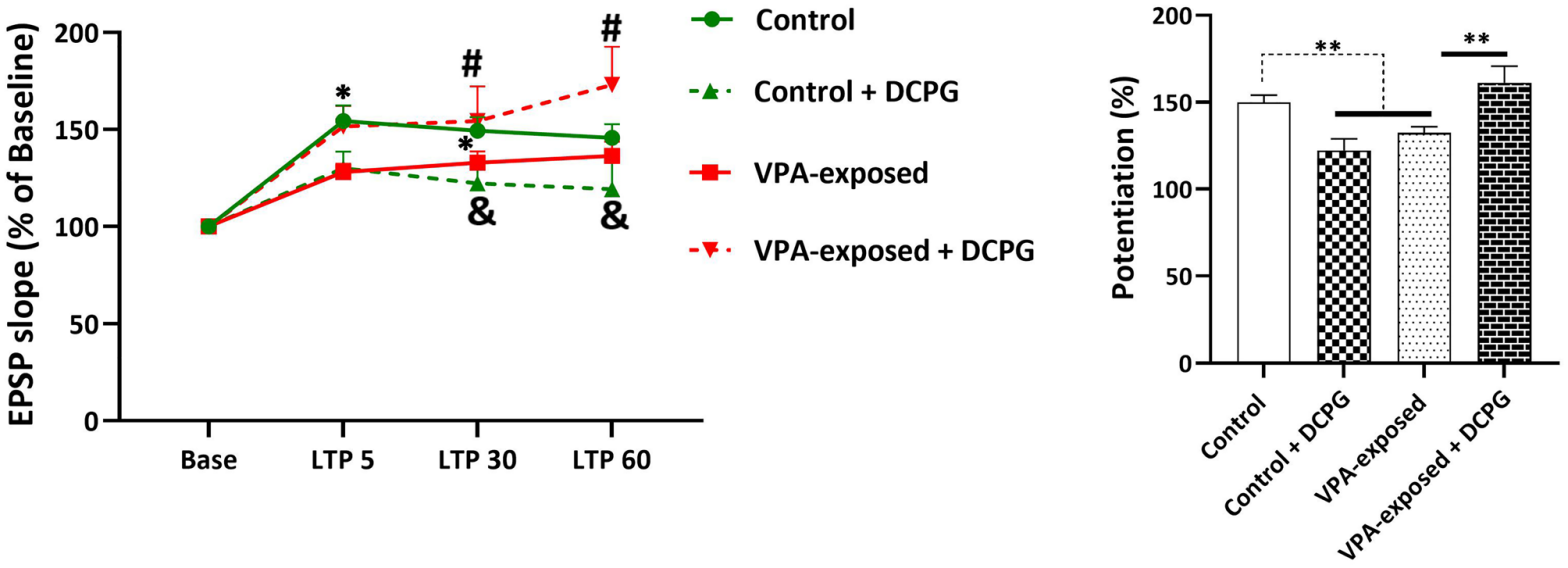
mGlu8 receptor agonist, (S)-3,4-DCPG, has opposite effects on long term potentiation in the dentate gyrus of control and VPA-exposed rats. Left panel shows fEPSP slope change (%) versus time following HFS in both groups. Right bar graphs show the average fEPSP slope potentiation during 60 min post-HFS. Data are reported as as mean ± SEM. In left panel: *P < 0.5 control versus VPA-exposed, # P < 0.5 VPA-exposed versus VPA-exposed + DCPG, & P < 0.5 Control versus Control + DCPG (two-way repeated-measures ANOVA). In right panel: **P < 0.01 (one-way ANOVA). The number of animals in each group for electrophysiological recordings was as follows: Control group: 14 rats, VPA-exposed group: 14 rats, Control + DCPG group: 8 rats, VPA-exposed + DCPG group: 8 rats.

LTP, evoked by HFS stimulation of the PP-DG pathway, was reduced by VPA-exposure. Representative examples of evoked field potential in the DG recorded prior to and 60 min after high-frequency stimulation are shown in Fig. 3. The results showed that prenatal exposure to VPA resulted in decreased fEPSP of DG granular neurons (Fig. 4). Two-way repeated-measures ANOVA results for slope of fEPSP of the granular cell of DG are as follows; time effect [F (1.277, 51.07) = 2.5702, P = 0.0340], group effect [F (3, 40) = 2.999, P = 0.0418] and interaction [F (6, 80) = 5.116, P = 0.0604]. Post-hoc comparisons indicated significant difference in different time points between experiment groups (left panel of Fig. 4.). Moreover, the average EPSP slope potentiation during the 60 min post-HFS was calculated for each group (right panel of Fig. 4). One-way ANOVA analysis for average EPSP slope potentiation showed significant differences between experimental groups (F (3, 136) = 8.145, P < 0.0001, One-way ANOVA). Consistent with our previous findings, VPA-exposed rats exhibited significantly less fEPSP slope LTP than control animals (P = 0.0018). Post-hoc comparisons indicated that intrahippocampal injection of mGlu8 receptor agonist, DCPG, has opposite effects in control and VPA-exposed rats. DCPG significantly inhibited LTP (decrease in slope of EPSP) at PP-DG pathway in control animals (P = 0.0053) but produced significant increase in slope of EPSP in VPA-exposed rats (P = 0.0035, Fig. 4).

### Effects of DCPG on field PS LTP in PP-DG pathway

**Figure 5:**
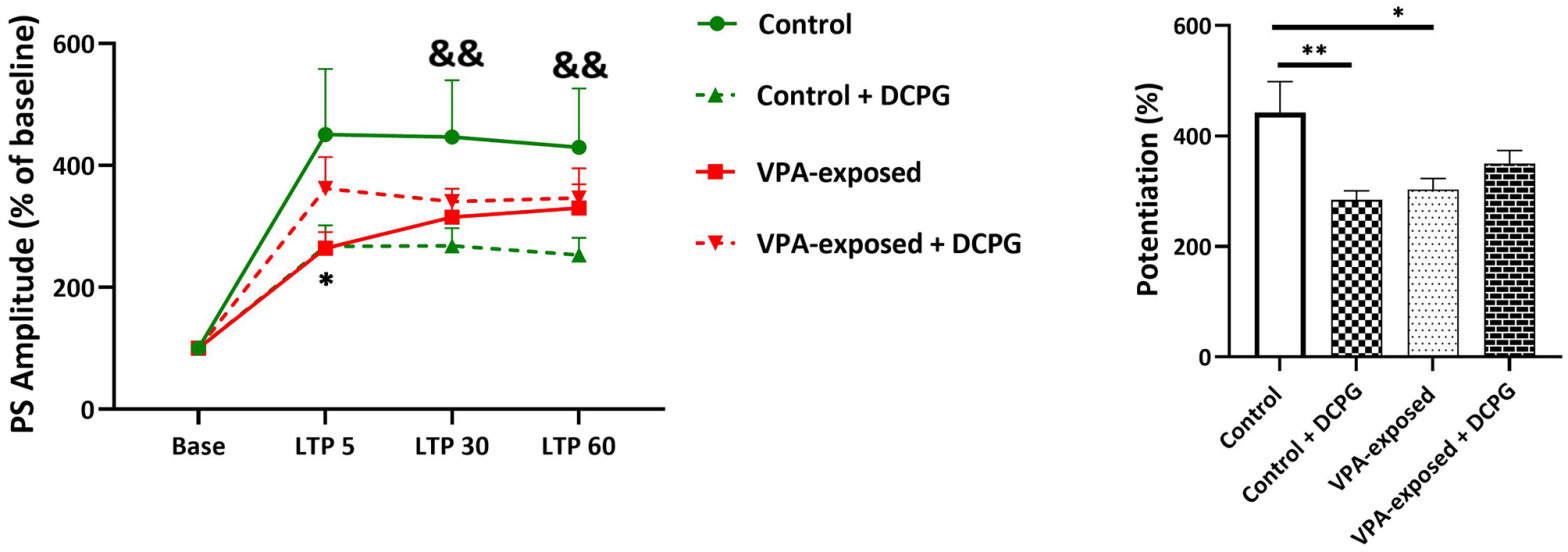
mGlu8 receptor agonist, (S)-3,4-DCPG, inhibited PS LTP at PP-DG pathway in control animals. Left panel shows PS amplitude change (%)versus time following HFS in both groups. Right bar graphs show the average PS amplitude potentiation during 60 min post-HFS. Two-way repeated-measures ANOVA, *P < 0.05, **P < 0.01. Data are reported as as mean ± SEM. In left panel: *P < 0.5 control versus VPA-exposed, && P < 0.01 Control versus Control + DCPG (two-way repeated-measures ANOVA). In right panel: *P < 0.05, **P < 0.01 (one-way ANOVA). The number of animals in each group for electrophysiological recordings was as follows: Control group: 14 rats, VPA-exposed group: 14 rats, Control + DCPG group: 8 rats, VPA-exposed + DCPG group: 8 rats.

Population spike LTP is shown in Fig. 5. Two-way repeated-measures ANOVA results for PS amplitude of the granular cell of DG are as follows; time effect [F (1.452, 56.64) = 0.1002, P = 0.8424], group effect [F (3, 40) = 1.467, P = 0.2386] and interaction [F (6, 80) = 1.391, P = 0.2289]. The average PS amplitude during the 60 min post-HFS was calculated for each group (right panel of Fig. 5). One-way ANOVA analysis for average PS amplitude showed significant differences between experimental groups (F (3, 136) = 4.704, P = 0.0037, One-way ANOVA). Consistent with our previous findings, VPA-exposed rats exhibited significantly less PS LTP than control animals (P = 0.0149). DCPG significantly inhibited PS LTP at PP-DG pathway in control animals (P = 0.0040). Although DCPG increased PS LTP in in VPA-exposed rats but this increase was not statistically significant (P > 0.5).

## Discussion

The present study showed that a VPA-induced rat model of autism alters glutamatergic neurotransmission, or more specifically, synaptic plasticity in rat hippocampus. The significant reduction of LTP suggests that ASD triggers substantial changes in glutamatergic transmission. To determine the mechanism underlying the effect of a VPA-induced rat model of autism on LTP, we used S-3,4-DCPG, a mGlu8 receptor agonist. We showed that intrahippocampal injection of mGlu8 receptor agonist, S-3,4-DCPG, has opposite effects in control and VPA-exposed rats. S-3,4-DCPG, significantly inhibited LTP at PP-DG pathway in control animals but produced significant increase in LTP in VPA-exposed rats. Moreover, the findings from our study highlight the significant impact of prenatal VPA exposure on social interaction behavior in rat offspring, consistent with previous reports. The administration of S-3,4-DCPG effectively reversed the decrease in SNPI induced by VPA exposure, underscoring the potential therapeutic efficacy of mGlu8 modulation in ameliorating social deficits associated with ASD. These findings contribute to our understanding of the complex neurobiology underlying social interaction impairments in ASD and suggest a role for mGlu8 receptors.

Group III mGlu receptors may function as autoreceptors on the terminals of PP afferents to dentate granule cells, according to previous electrophysiological studies that showed a selective agonist for group III metabotropic glutamate receptors (mGlu 4/6/7/8) can suppress fEPSPs evoked by PP stimulation^42–44^. Furthermore, intracerebral injection of a group III mGlu receptor agonist has been shown to significantly inhibit LTP in the DG of freely moving rats^45^. Immunohistochemical evidence has demonstrated that several types of groups III mGlu receptors are localized to the molecular layer of the DG^27,46,47^. Among these receptor subtypes, mGlu8 receptors display the most prominent expression in the presynaptic elements in the molecular layer of the dentate gyrus, implicating this mGlu receptor subtype as the primary group III mGlu receptor responsible for modulation of PP synapses^27,44,46,47^.

Results from the present experiments support this hypothesis by demonstrating that activation of mGlu8 receptors by (S)-3,4-DCPG inhibits LTP at PP-DG pathway in control animals. Jin et al., also reported that PP-evoked fEPSPs were suppressed in control slices by the (S)-3,4-DCPG^48^. Also it has been shown that DCPG inhibits fEPSPs in the PP of Crl:Wi rats^29^. At the PP-DG synapses in the hippocampus, activation of mGlu8 also results in chemical LTD, a common type of synaptic plasticity^49^.

The mGlu8 receptor, which mainly functions as an autoreceptor to prevent further glutamate release, is hypothesized to reside in presynaptic terminals^27,48^. As a Gi/o-coupled autoreceptor, mGlu8 inhibits glutamate release to maintain homeostasis of glutamatergic transmission. Related studies have shown that, mice lacking the mGlu8 receptor showed enhanced LTP in the hippocampus, suggesting that the receptor may act as a negative regulator of LTP in hippocampus^29^. Moreover it has been reported that mGlu8 receptor activation provides a powerful inhibitory control of synaptic transmission within the lateral amygdala^50^. Additionally, they demonstrated that LTP induced by tetanic stimulation was completely inhibited by (S)-3,4-DCPG. This shows that (S)-3,4-DCPG may inhibit synaptic transmission to dentate gyrus pyramidal neurons. In this regard Susanne and Markus reported that (S)-3,4-DCPG inhibit synaptic transmission not only to principal neurons, but also to inhibiting interneurons^50^.

Additionally, VPA enhances inhibitory GABAergic neurotransmission and increases inhibitory post synaptic potential^51^. Increased GABAA-mediated hyperpolarizing response block NMDA receptors, preventing the induction of LTP^52^. In the meantime, mGlu8 receptors may function as a heteroreceptor to block the release of non-glutamatergic transmitters like GABA^53,54^. So, we can conclude that mGlu8 receptors activation by decreasing the GABA_A_-mediated response facilitates the induction of LTP in VPA-exposed animals.

Although the mechanisms that underlie the effects of ASD on LTP are not well-understood, the present study demonstrated that changes in mGlu 8 receptors may are one of mechanisms underlie the effects of VPA-induced LTP impairment. In Conclusion the present study suggests that prenatal VPA-exposure can impair hippocampal LTP, and mGlu 8 receptors inhibition maybe have stimulatory effects on LTP induction in the DG.

## Conclusion

In conclusion, our study advances our understanding of the complex relationship between glutamatergic neurotransmission, synaptic plasticity, and ASD in a VPA-induced rat model. The observed alterations in LTP and the contrasting effects of the mGlu8 receptor agonist, S-3,4-DCPG, provide valuable insights into the underlying neurobiology of autism. These findings encourage further exploration into the potential of mGlu8 receptors as therapeutic targets and highlight the importance of glutamatergic neurotransmission in addressing the diverse manifestations of ASD.

## Data Availability

Data will be made available on reasonable request from corresponding author.
